# Immunometabolic Stress and Immune Suppression in Clear-Cell Renal Cell Carcinoma: Perspectives in Therapeutic Strategy

**DOI:** 10.3390/ijms27136021

**Published:** 2026-07-04

**Authors:** Tuong-Vi Nguyen, Tien Hsu

**Affiliations:** 1 Department of Biomedical Sciences and Engineering, National Central University, 300 Zhongda Rd., Taoyuan 320317, Taiwan; 108886602@cc.ncu.edu.tw; 2 Graduate Institute of Biomedical Sciences, China Medical University (Taiwan), 91 Hsueh-Shih Rd., Taichung 404333, Taiwan

**Keywords:** clear-cell renal cell carcinoma, hypoxia, immunometabolism, metabolic reprogramming, immune suppression, kynurenine pathway

## Abstract

Solid tumors frequently experience hypoxia during tumor progression, resulting in profound metabolic alterations. This phenomenon is particularly pronounced in clear-cell renal cell carcinoma (ccRCC) because of loss of the von Hippel–Lindau (*VHL*) tumor suppressor gene and constitutive activation of hypoxia-inducible factor (HIF) signaling. ccRCC is the most common subtype of kidney cancer, and durable therapeutic responses remain limited despite advances in immune checkpoint inhibition. Owing to its strong pseudohypoxic phenotype and extensive metabolic rewiring, ccRCC is widely regarded as a metabolic disease. These alterations generate a unique immune landscape characterized by abundant immune-cell infiltration together with profound T-cell dysfunction and exhaustion. This paradoxical “immune-hot yet immunosuppressed” phenotype is largely driven by hypoxia-associated immunometabolic reprogramming within tumor cells and the tumor microenvironment (TME). Several metabolic pathways are critically involved in this process, including lactate acidosis, arginine (Arg) depletion, tryptophan (Trp) depletion, kynurenine (Kyn)-mediated T-cell exhaustion, and adenosine-driven immune suppression. This review summarizes the current understanding of hypoxia-driven immunometabolic interactions in ccRCC and discusses how targeting these pathways may improve future therapeutic strategies against this aggressive malignancy.

## 1. Introduction

Clear-cell renal cell carcinoma (ccRCC) is the most prevalent subtype of renal cell carcinoma, occurring in up to 80% of all RCC cases, and its incidence and death rates continue to rise [1,2]. Multiple therapies are now available, including immune checkpoint inhibitors (ICIs), tyrosine kinase inhibitors (TKIs), and Hypoxia-inducible factor (HIF)-2α inhibitors. Although these therapeutics have improved outcomes, durable responses remain limited, and resistance is common. For example, anti-angiogenesis therapies based on TKI treatment are frequently limited by the development of acquired resistance, which remains a major obstacle to long-term disease control [3,4]. The more recent ICI therapy initially presented only a ~30% response rate [5,6,7]. However, there have been significant advances in more recent therapeutic strategies. In an open-label, phase 3 trial, pembrolizumab–axitinib combination showed median progression-free survival (PFS) at 15.1 months with an objective response rate (ORR) of 59.3% vs the sunitinib treatment group showing median PFS at 11.1 months and an ORR of 35.7% [8]. Another phase 3 trial compared nivolumab plus ipilimumab with sunitinib for previously untreated advanced ccRCC. It showed the ORR at 42% versus 27%, and the complete response (CR) rate at 9% versus 1%. The median PFS was 11.6 months versus 8.4 months [7]. Nonetheless, the optimal ICI regimen and the selection of target patient groups still require improvement. Potential long-term side effects, resistance, and variable responses of HIF-2α inhibitor treatment are also of concern.

A majority (up to 80%) of the ccRCC cases is driven by the loss of the *von Hippel-Lindau* (*VHL*) tumor suppressor gene [9,10]. *VHL* encodes the substrate-recognition component of an E3 ubiquitin ligase that degrades the alpha subunits of HIF (HIF-1α, HIF-2α, and HIF-3α) [11]. Loss of *VHL* function therefore causes HIF stabilization even in the normoxic conditions, thereby inducing metabolic reprogramming such as a shift from oxidative phosphorylation (OXPHOS) to glycolysis and altered amino acid metabolism [12].

ccRCC exhibits a distinctive immunometabolic phenotype among solid tumors. It is typically classified as “immune-hot,” characterized by abundant infiltration of CD8^+^ T cells, macrophages, and other effectors such as natural killer cells (NKs) and CD4^+^ Th1 cells [13]. However, the cancer remains highly immunosuppressive and resistant to effective immune control. Recent advances in cancer metabolomics have provided tangible mechanistic insights into this apparent paradox.

In this review, we will focus on the role of immunometabolic dysregulation induced by the ccRCC cells and the reconstituted microenvironment, since this knowledge can inform a logical design of amenable therapeutic strategies.

## 2. Clear-Cell Renal Cell Carcinoma (ccRCC) Is Immune-Hot Yet Immunosuppressed

ccRCC is one of the most immune-infiltrated solid tumors, yet paradoxically exhibits profound immune suppression, with abundant immune infiltration correlating with poor prognosis and limited response to immune checkpoint inhibitors [14,15]. This “immune-hot but immunosuppressed” phenotype is a defining feature of ccRCC [16,17,18]. Single-cell RNA sequencing further revealed that, although CD8^+^ T cells are abundant, disease progression is accompanied by accumulation of terminally exhausted T cells and immunosuppressive tumor-associated macrophages (TAMs), which correlate with adverse clinical outcomes [19].

Renal lymphatics are concentrated in the kidney cortex, where most ccRCCs arise, and lymphangiogenesis is further enhanced by tumor-derived VEGF-C [1,20,21,22]. It has been hypothesized that, since lymphatics can recruit immune cells into the surrounding tissue, the cortically enriched lymphatic network may contribute to the prominent immune infiltration observed in ccRCC. Because lymphatic endothelial cells can express immunosuppressive molecules such as PD-L1, they may then impair T-cell activation before tumor entry [23]. However, spatial transcriptomic analyses have shown that peritumoral tertiary lymphoid structures (TLSes) at the tumor–normal interface harbor immunosuppressive niches enriched with regulatory T cells, suggesting that immune suppression occurs both before and after immune cell infiltration [24,25,26]. As such, whether the cortical distribution of renal lymphatics directly drives the immune-hot yet immunosuppressed phenotype of ccRCC remains unresolved. Other immunosuppressive mechanisms likely also play important roles.

## 3. Metabolic Reprogramming Is a Key Driver of Immune Suppression

The *VHL* deficiency–HIF axis plays a pivotal role in the pathogenesis of ccRCC, in which biallelic inactivation of the *VHL* tumor suppressor gene leads to constitutive stabilization of HIF-1α and HIF-2α under normoxic conditions, a state termed pseudohypoxia [11,12]. The dysregulated oxygen sensing mechanism orchestrates profound metabolic changes. This shifts cellular energetics toward aerobic glycolysis, enhanced lipid biosynthesis and accumulation, and activation of alternative amino acid metabolic pathways. As such, ccRCC has been recognized as a metabolic disease [1,27,28]. These changes fuel tumor proliferation, survival under stress, and lymphangio/angio-genesis. They also generate an immunosuppressive tumor microenvironment (TME) through mechanisms such as lactate-induced acidosis, Arginine (Arg) depletion, Tryptophan (Trp) depletion, kynurenine (Kyn)-mediated T-cell exhaustion, and adenosine-driven immune suppression.

### 3.1. Generation of Immunosuppressive Tumor Microenvironment (TME)

The metabolic outputs of HIF-mediated reprogramming create a highly nutrient-deprived, metabolite-rich TME that impairs immune surveillance. Such changes not only induce CD8^+^ T-cell exhaustion but also promote induction of M2-type (anti-inflammatory) TAMs, Tregs, and myeloid-derived suppressor cells (MDSCs). At least five major metabolic pathways are involved in effecting an immunosuppressive TME.

### 3.2. Lactate Accumulation and Acidosis

**Figure 1:**
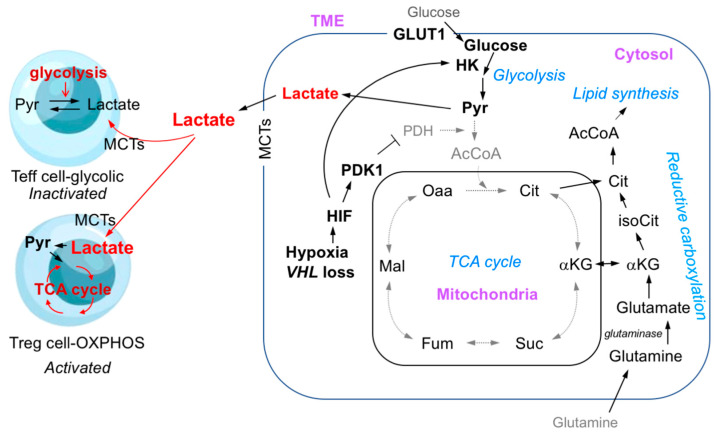
Lactate accumulation and acidosis. In clear-cell renal cell carcinoma (ccRCC) cells, loss of *VHL* function leads to hypoxia-inducible factor (HIF) upregulation, PDK1 overexpression, and inhibition of pyruvate conversion to acetyl CoA (AcCoA), causing reduction of Tricarboxylic acid (TCA) cycle activity in the mitochondria. Lack of TCA cycle activity results in the use of glutamine to generate lipids in a process named reductive carboxylation (glutamine-glutamate-αKG-isocitrate-citrate-AcCoA). The cytosolic pyruvate level is further increased because of the upregulation of Glut1 and hexokinase (HK) by HIF, promoting glycolysis. Pyruvate is then converted to lactate, and lactate is secreted into the microenvironment, causing acidosis. AcCoA, Acetyl CoA; αKG, α-Ketoglutarate; Cit, Citrate; Fum, Fumarate; HK, Hexokinase; isoCit, Isocitrate; Mal, Malate; Oaa, Oxaloacetate; PDH, Pyruvate dehydrogenase; PDK1, Pyruvate dehydrogenase kinase 1; Pyr, Pyruvate; Suc, Succinate. The illustration was generated by the authors but used stock images of immune cells from BioRender. Hsu, T. (2026) https://BioRender.com/pu6xpwq (accessed on 29 June 2026).

HIF promotes a shift to aerobic glycolysis by increasing glucose transport via inducing expression of glucose transporter Glut1 [29,30] and by inducing expression of glycolytic enzymes such as hexokinases (HKs) [31]. Glycolysis generates pyruvate, which in normal physiological condition enters the Tricarboxylic Acid (TCA) cycle in the mitochondria. This process requires pyruvate dehydrogenase (PDH) for converting pyruvate into acetyl CoA (AcCoA). In hypoxic and pseudohypoxic conditions, activated HIF induces expression of pyruvate dehydrogenase kinase 1 (PDK1) that phosphorylates and inhibits PDH, thus preventing pyruvate conversion to AcCoA and entry into the TCA cycle (Figure 1) [32,33]. Reduced TCA cycle activity results in reductive carboxylation, which utilizes glutamine to generate lipids [glutamine-glutamate-αketoglutarate (αKG)-isocitrate-citrate-AcCoA) [28,32,34]. The combined effects result in significantly increased cytosolic pyruvate accumulation and subsequent lactic acid production. While this metabolic switch is observed in most hypoxic solid tumors, in ccRCC it is exacerbated because of the *VHL* loss, creating a hypoxic-like niche that further amplifies glycolysis even in normoxia [12,35].

A comprehensive metabolomic study showed that in ccRCC tumor samples the metabolites in the upper glycolysis, including glucose, glucose-6-phosphate, and fructose-6-phosphate, as well as the end product of glycolysis, lactic acid, are significant increased compared with normal kidneys [28]. Furthermore, the citrate levels are increased while the isocitrate levels are decreased. This is in line with the previous cell-based study that demonstrated reduced TCA cycle activity but increased reductive carboxylation pathways in ccRCC [32]. This metabolic signature is unique to ccRCC as compared with all other cancers [28].

The over-produced lactic acid is released from the tumor cells via monocarboxylate transporters (MCTs), lowering the pH of the microenvironment (often to pH 6.0–6.5). Siska et al. demonstrated that in ccRCC, exhaustion of CD8^+^ tumor-infiltrating lymphocytes was induced by acidosis and nutrient stress [36]. Conversely, lactate acidosis promotes Treg function and stability, further exacerbating immune suppression, particularly in ccRCC [37].

The opposing effects of acidosis on effector and Tregs stem from lineage-intrinsic metabolic and transcriptional programs in the two T-cell types. Effector T cells (Teffs) depend on glycolysis, which produces pyruvate and lactate, for short bursts of effector functions requiring rapid energy output. Excess lactate directly inhibits this pathway because of the backflow of the end products (lactate to pyruvate) of glycolysis [38,39]. Tregs, on the other hand, are metabolically flexible, relying on OXPHOS, fatty acid oxidation, and, importantly, alternative fuels such as lactate itself. For example, Tregs express MCT1 that imports lactate as an alternative carbon source for gluconeogenesis and TCA cycle anaplerosis—especially in glucose-poor, lactate-rich TME [40]. In addition, the Treg marker FOXP3 actively suppresses glycolysis while promoting OXPHOS by increasing lipid β-oxidation and upregulation of components of the electron transport complexes. Such metabolic adaptability renders Tregs resistant to (or even thriving in) nutrient-stressed, acidic conditions [41,42,43].

### 3.3. Arginine (Arg) Depletion

Arg depletion is driven by a combination of tumor-intrinsic metabolic defects (urea cycle downregulation) and extrinsic immune cell activity [high arginase-1 (ARG1) expression in MDSCs and TAMs], creating a hostile environment for Teffs.

**Figure 2:**
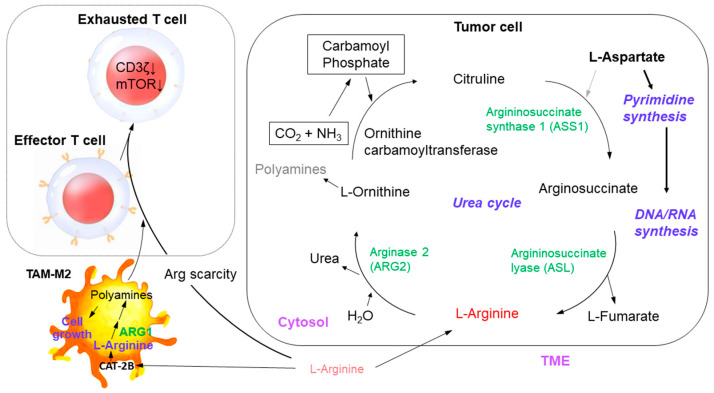
Urea cycle downregulation and Arg depletion. In ccRCC cells, Argininosuccinate synthase 1 (ASS1) and Argininosuccinate lyase (ASL) are downregulated, preventing aspartate (Asp) from entering the urea cycle. Concurrently, arginine (Arg) production is reduced, forcing the cancer cells to absorb Arg from the microenvironment, contributing to Arg scarcity in the TME. The Arg scarcity is exacerbated by the action of M2-type tumor-associated macrophages (TAMs) [as well as myeloid-derived suppressor cells (MDSCs)] that actively import Arg by CAT-2B. Arg scarcity also inactivates effector T cells (Teffs), causing exhaustion. The illustration was generated by the authors but used stock images of immune cells from BioRender. Hsu, T. (2026) https://BioRender.com/pu6xpwq (accessed on 29 June 2026).

Normally, the urea cycle processes the nitrogen wastes from amino acid catabolism in humans, converting toxic ammonium (NH_4_^+^)-containing molecules into non-toxic urea for excretion by the kidneys [44] (Figure 2). Note that the urea cycle is active mostly in the liver and, to a lesser extent, in the kidney. The major function of the urea cycle in the kidney includes net production of Arg. Kidney-produced Arg is disseminated to other parts of the body, which constitutes a major source (up to 50%) of Arg for other cell types [45,46]. Kidney tubule cells do express ARG2 in the mitochondria that can break down Arg to form urea and ornithine. However, this serves as a modulator of the Arg levels and for generating limited amounts of polyamines important for DNA stabilization [47,48]. In addition, the urea generated in renal urea cycle helps establish the corticomedullary osmotic gradient essential for maximum water reabsorption and urine concentration [49].

Significantly, the ccRCC cells downregulate the key urea cycle enzymes that produce Arg, argininosuccinate synthase 1 (ASS1) and argininosuccinate lyase (ASL) (Figure 2) [28,50,51], making the ccRCC cells auxotrophic for Arg (unable to synthesize Arg endogenously). Since the urea cycle consumes aspartate (Asp) to form argininosuccinate and Arg, in sequential reactions catalyzed by ASS1 and ASL, downregulation of these two enzymes spares Asp. It is hypothesized that the spared Asp is then shunted into the aspartate-malate shuttle and pyrimidine production, which is essential for nucleic acid synthesis in rapidly dividing tumor cells [50].

Thus, downregulation of the urea cycle forces ccRCC cells to import Arg from the extracellular space for protein synthesis and for preventing mTOR inhibition, since Arg scarcity is a major checkpoint for mTOR signaling [52,53,54]. The net result is increased competition for Arg in the TME. Furthermore, TAMs and MDSCs express high levels of ARG1 and the cationic amino acid transporter CAT-2B, further depleting extracellular Arg (Figure 2) [55,56,57]. ARG1 converts Arg to ornithine, which is then converted to polyamines (putrescine, spermidine, and spermine). Polyamines (highly positively charged molecules) act as stabilizers of DNA (highly negatively charged) and intracellular pH buffers in the acidic TME, thus preventing cell death in myeloid suppressor cells [47,48].

In the effector cells, Arg deficiency leads to downregulation of T-cell receptor CD3ζ chain, since CD3ζ mRNA stability is sensitive (a checkpoint) to metabolic stress caused by Arg starvation [58,59]. In addition, Arg depletion can reduce mTOR activation, because normally mTOR activation is mediated by Arg binding to the sensor proteins CASTOR1 (cytosolic) and SLC38A9 (lysosomal) [52,53,54]. Arg depletion also increases the level of uncharged tRNAs, leading to GCN2 activation, global reduction of translation efficiency, and Teff cell cycle arrest [60,61].

In normal renal tubular cells, conversion of Arg to ornithine by mitochondrial ARG2 is a tightly regulated metabolic process. The resulting ornithine is metabolized by mitochondrial ornithine aminotransferase (OAT) to generate glutamate and pyrroline-5-carboxylate (P5C), which are subsequently converted to αKG to replenish the TCA cycle [49]. In contrast, the defective TCA cycle in ccRCC reduces αKG utilization, making downregulation of mitochondrial ARG2 essential to prevent excessive ornithine and polyamine accumulation [62]. Teffs, however, rely on extracellular Arg for activation and proliferation. Consequently, Arg depletion in the ccRCC TME arises through two complementary mechanisms, tumor cell-intrinsic suppression of ASS1/ASL, which increases tumor cells’ dependence on extracellular Arg, and tumor-extrinsic consumption of Arg by TAMs and MDSCs, which overexpress the arginine transporter CAT-2B and cytosolic ARG1.

The relative contributions of tumor-intrinsic ASS1/ASL silencing and myeloid ARG1-mediated arginine depletion remain unknown because of substantial interpatient heterogeneity and the spatial complexity of the tumor microenvironment. Nevertheless, their biological importance can be inferred from their prevalence. ASS1/ASL silencing is a widespread clonal feature of ccRCC. Large-scale TCGA-based multiomic analyses indicate that approximately 85–90% of primary ccRCC tumors exhibit marked transcriptional downregulation or complete silencing of ASS1 and ASL [51,63]. This metabolic phenotype is closely associated with *VHL* loss and chronic HIF activation, creating a pervasive state of Arg auxotrophy throughout the tumor mass [50,64]. In contrast, ARG1 expression is largely confined to subsets of MDSCs and M2-like TAMs, as demonstrated by single-cell transcriptomic analyses of human ccRCC cohorts, including GSE159115 [64,65]. Rather than representing a tumor cell-intrinsic alteration, myeloid ARG1 establishes localized enzymatic niches that deplete extracellular Arg in regions of dense immune infiltration, thereby reinforcing immunosuppression.

### 3.4. Tryptophan (Trp) Depletion

**Figure 3:**
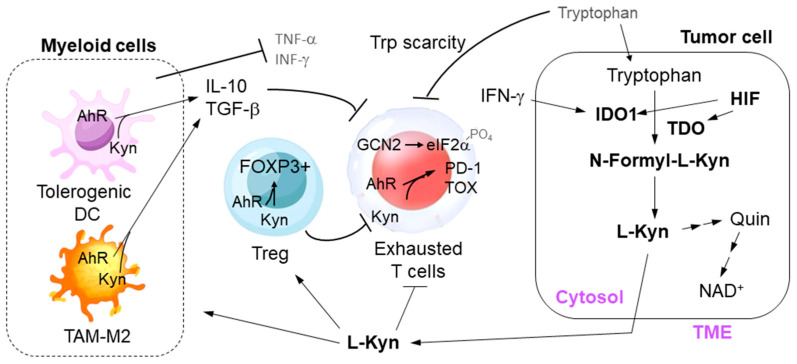
Tryptophan (Trp) depletion and kynurenine (Kyn)-mediated T-cell exhaustion. While ccRCC cells overexpress indoleamine 2,3-dioxygenase 1 (IDO1) and Trp 2,3-dioxygenase (TDO), inflammatory cell-produced IFN-γ also induces the expression of IDO1, which leads to overproduction of Kyn, causing Trp depletion in the microenvironment. Trp scarcity causes T-cell exhaustion via GCN2-induced eIF2α phosphorylation and global downregulation of translation. Concurrently, overproduced Kyn is absorbed by the immune cells, binds to AhR, and induces different cellular responses in different immune cells. The illustration was generated by the authors but used stock images of immune cells from BioRender. Hsu, T. (2026) https://BioRender.com/pu6xpwq (accessed on 29 June 2026).

In hypoxic conditions, tumor and stromal cells upregulate indoleamine 2,3-dioxygenase 1 (IDO1) and Trp 2,3-dioxygenase (TDO), which catabolize essential amino acid Trp into Kyn and downstream metabolites (Figure 3) [66]. Increased Kyn is used for producing additional NAD^+^, a key driver of energy metabolism critical for cell growth [67]. This tumor-skewed process rapidly depletes extracellular Trp, often to levels insufficient for T-cell needs [66,68]. The process is tumor-intrinsic and inflammation-amplified [69,70]. That is, IDO1 and TDO are upregulated in cancer cells themselves, independent of external inflammation, leading to increased Trp catabolism and Kyn production. On the other hand, IFN-γ secreted by activated T cells also strongly induces IDO1 expression in tumor cells, stromal cells, and myeloid cells as a counter-regulatory mechanism to dampen excessive inflammation and T-cell responses [71,72]. Reduced Trp levels in Teffs activate the GCN2 kinase. GCN2 is an amino acid sensor that responds to uncharged tRNAs accumulating in Trp (or Arg) scarcity. Upon activation, GCN2 phosphorylates eukaryotic initiation factor 2α (eIF2α) at serine 51, leading to global downregulation of translation, cell cycle arrest in G1 phase, impaired cytokine production (e.g., IL-2 and IFN-γ), and induction of anergy (unresponsiveness to antigen stimulation) or apoptosis in CD4^+^ and CD8^+^ Teffs [61,73,74]. In ccRCC and other cancers, this contributes to immune evasion by limiting Teff responses while favoring Tregs that are less sensitive to GCN2 activation because of the higher capacity in metabolic adaptations [75]. In myeloid cells, GCN2 is important for the polarization of immunosuppressive TAMs and activation of MDSCs [76]. Trp scarcity is further exacerbated in ccRCC by the heightened HIF activation, leading to increased tumor cell competition for Trp, thus limiting Teff functions even before tumor tissue hypoxia sets in [13,77].

### 3.5. Kynurenine (Kyn)-Mediated T-Cell Exhaustion

The accompanying effect of Trp depletion is Kyn overproduction by the cancer cells, leading to accumulation in the extracellular space [78]. The Kyn pathway is a negative feedback mechanism that resolves inflammation [71,72], in which Kyn overproduced by inflamed or cancer tissues suppresses the activity of Teffs. In ccRCC, the Kyn pathway hyperactivation is tumor-intrinsic (e.g., via IDO1 and TDO upregulation in cancer cells) and immune cell-induced (e.g., IFN-γ-induced IDO1 expression), as described above (Figure 3). Mechanistically, Kyn binds and activates the aryl hydrocarbon receptor (AhR) in CD8^+^ T cells, promoting transcriptional changes that upregulate exhaustion markers (e.g., PD-1 and TOX) and suppress metabolic fitness (e.g., reduced glycolysis and fatty acid catabolism) [79]. In dendritic cells (DCs) and TAMs, the Kyn-AhR activity increases production of immunosuppressive factors TGF-β and IL-10, while suppressing inflammatory factors TNF-α and INF-γ in several non-ccRCC solid tumors [80,81]. In melanoma, the Kyn-AhR signaling also induces FOXP3 expression, favoring Treg differentiation over effector responses [82]. In ccRCC, high Kyn/Trp ratios correlate with advanced cancer stages, poor PFS, and reduced survival [83]. In ccRCC treatment models, Kyn exposure directly upregulates PD-1 and TOX in T cells [84,85]. As such, prognostic models based on Kyn pathway genes or exhaustion signatures (e.g., chemokines influenced by the Kyn levels) can predict survival and therapy response in ccRCC [13,83].

In preclinical non-ccRCC cell and animal models, the growth of tumors enriched in Trp catabolites is blocked by AhR inhibitors alone or AhR inhibitors in combination with anti-PD-1 antibody [80,86].

### 3.6. Adenosine-Driven Immune Suppression

**Figure 4:**
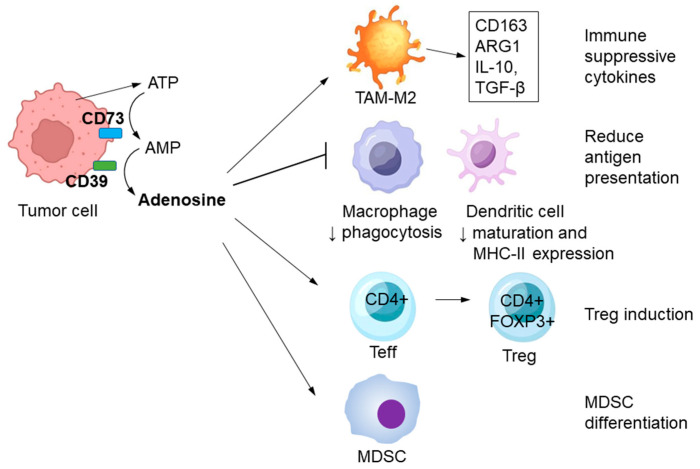
Adenosine-driven immune suppression. Dying or stressed cells release ATP, which is catabolized by the sequential actions of two ectonucleotidases CD73 and CD39 on the cancer cell surface. The resulting adenosine interacts with adenosine receptors on various immune cells, inducing cellular responses that, in aggregate, lead to immune suppression. The illustration was generated by the authors but used stock images of immune and tumor cells from BioRender. Hsu, T. (2026) https://BioRender.com/pu6xpwq (accessed on 29 June 2026).

Dying or stressed tumor cells release ATP primarily as a damage-associated molecular pattern (DAMP) to signal danger and trigger immune responses. DAMP is not random leakage but a regulated process, especially during immunogenic cell death. Extracellular ATP can recruit and activate antigen-presenting cells (especially DCs) via purinergic receptors (mainly P2X7 and P2Y2) [87,88]. However, the immunogenic ATP can be coerced to dampen the immune response (Figure 4). The extracellular ATP is rapidly converted to adenosine by two ectonucleotidases expressed on the surface of cancer and other stromal cells such as Tregs, MDSCs, and M2-type TAMs: CD39 (also known as NTPDase1) converts ATP to AMP, and CD73 (also known as 5′-nucleotidase) converts AMP to adenosine [89,90]. Both enzymes are upregulated by HIF-1α/HIF-2α, leading to markedly elevated extracellular adenosine levels [91]. Indeed, it has been shown that CD73-mediated adenosine production contributes to immune suppression and poor prognosis in RCC patients [92]. On the other hand, TAMs, MDSCs, and Teffs highly express the high-affinity A2A receptor (A2AR) that responds to low levels of adenosine (250–700 nM), and TAMs and MDSCs also express the low-affinity A2B receptor (A2BR) that responds to high levels of adenosine (up to 25 μM) [93]. These G-protein-coupled P1 purinergic receptors increase intracellular cAMP upon adenosine binding, activating protein kinase A (PKA) and downstream signaling (e.g., CREB and EPAC) [94,95,96]. Importantly, increased expression of A2AR in metastatic RCC tissue has been linked to resistance to standard TKI and ICI therapies [97]. The study also showed that increased A2AR expression is largely located within the tumor-infiltrating lymphocytes, while in some severe metastatic cases, RCC cells can also overexpress A2AR.

Adenosine signaling through A2AR and A2BR drives profound immunosuppression: (1) The signaling induces polarization of TAMs toward an M2-like phenotype with increased expression of CD163, ARG1, IL-10, and TGF-β [98]. (2) The receptor activation impairs antibody-mediated macrophage phagocytosis, and suppresses DC maturation and MHC-II expression, thus reducing antigen presentation [99,100]. (3) The signaling reduces production of pro-inflammatory cytokines (e.g., IL-12 and TNF-α) produced by DCs, macrophages, and neutrophils that recruit Teffs and NK cells [98,99,101] while inducing FOXP3 expression in CD4^+^ T cells, leading to Treg development [102]. (4) The signaling enhances recruitment and activation of MDSCs [99,103]. These studies are not ccRCC-specific but represent general immune cell properties that should be applicable to ccRCC-associated immune responses.

Interestingly, Clayton et al. discovered that exosomes isolated from mesothelioma patients express CD73 and CD39 [104]. Because exosomes can rapidly disseminate through lymphatics to lymph nodes, their expression of CD73 and CD39 may contribute to systemic immunosuppression and pre-metastatic niche formation [105]. Comparative evidence has not yet been demonstrated in ccRCC [106] but may worth further investigation.

### 3.7. Vascular Involvement

ccRCC is characterized by abundant abnormal blood vessels driven by *VHL* loss resulting in HIF activation and VEGF overexpression. These vessels are tortuous, leaky, and poorly organized, resulting in increased extravasation of plasma fluid into the interstitial space [107]. This leads to elevated interstitial fluid pressure (IFP)—often 10–40 mmHg over atmospheric pressure in tumors vs. no increase in normal tissues—and a large volume of interstitial fluid (an edema-like state) [108,109]. Elevated IFP compresses blood vessels and limits convective transport, reducing delivery of oxygen, glucose, and other nutrients while impairing metabolite clearance (e.g., lactate and Kyn). Consequently, the effector cells are more prone to a shift to inefficient metabolic pathways, to accumulate reactive oxygen species, experience amino acid depletion, and undergo exhaustion or anergy [109,110,111].

Analysis of the tumor interstitial fluid of ccRCC indeed showed that it was poor in nutrients but rich in immune suppressive toxins such as Kyn [13,112,113]. As immune cells travel through the lymphatics toward the tumor, they must pass through this tumor-reconstituted interstitial fluid, contributing to suppression of immune cells.

This understanding also reinforces the notion that the goal of anti-angiogenic therapy should be normalizing the tumor-associated vasculature rather than eliminating it [114,115,116].

### 3.8. Why Immunotherapy Fails Despite Immune Infiltration

Spatial transcriptomic analyses of metastatic ccRCC revealed marked intratumoral heterogeneity in immune microenvironments, with distinct immune niches associated with either responsiveness or resistance to immune checkpoint inhibition. These findings highlight the complex spatial architecture of the immunosuppressive TME [18,25,26]. One of the major unresolved issues in ccRCC immunotherapy is the observation that tumors with abundant immune-cell infiltration often exhibit only modest and non-durable responses to immune checkpoint inhibition. A possible explanation is that although checkpoint blockade can partially restore inhibitory signaling pathways in exhausted T cells, it does not fully reverse the severe metabolic abnormalities within the TME. In ccRCC, persistent pseudohypoxia, intense nutrient competition, lactate-driven acidosis, and accumulation of immunosuppressive metabolites such as Kyn and adenosine collectively create a metabolically unfavorable environment that compromises the function of tumor-infiltrating lymphocytes. As a result, despite transient restoration of T-cell activation following PD-1/PD-L1 inhibition, effector cells often remain metabolically impaired and fail to maintain long-term anti-tumor activity.

## 4. Therapeutic Outlook

To counter the metabolic stresses that sabotage the anti-tumor immunity, efforts are being made to develop therapeutics that block the tumor’s metabolic interference. Nonetheless, current therapies addressing immunometabolic stress in ccRCC remain limited in scope, with only one class—HIF-2α inhibition—successfully translating the core *VHL* deficiency-driven metabolic reprogramming into approved clinical use. Inhibiting downstream elements such as IDO1 and/or TDO for counteracting Trp/Kyn pathway, or inhibiting arginases for counteracting Arg depletion, has shown preclinical promise but limited success in clinical settings. It should be noted that the effectiveness of anti-HIF therapeutics most likely can also be attributed to inhibiting other HIF-regulated hypoxic responses such as angiogenesis, epithelial-to-mesenchymal transition, and extracellular matrix remodeling. Nonetheless, addressing specific metabolic pathways in combination with anti-HIF therapeutics should be considered. In this section, we describe in details the clinical uses of HIF-2 inhibitor belzutifan. Other potential metabolic pathway modulators are summarized in Table 1.

### 4.1. HIF-2α Inhibitors

In a phase 3 trial (LITESPARK-011, NCT04586231), belzutifan plus lenvatinib was compared with cabozantinib in advanced renal cell carcinoma after anti-PD-1/PD-L1 therapy. The results showed median PFS of 14.8 months (belzutifan + lenvatinib) vs. 10.7 months (cabozantinib); median overall survival (OS) of 34.9 months vs. 27.6 months; and ORR of 52.6% (including ~5.4% CR) vs. 40.2% (including ~1.1% CR) [117].

In a more recent phase 3 trial (LITESPARK-005, NCT04195750), belzutifan vs. everolimus was compared in advanced ccRCC patients who had received ICI and antiangiogenic therapies. Belzutifan showed a significant benefit over everolimus with respect to PFS (24.0% vs. 8.3% at 18 months) and objective response (21.9% vs. 3.5% at 18 months). However, belzutifan did not demonstrate a statistically significant OS benefit. At the second interim analysis (median follow-up, 25.7 months), median OS was 21.4 months with belzutifan versus 18.1 months with everolimus (hazard ratio for death, 0.88; 95% CI, 0.73–1.07; two-sided *p* = 0.20). The estimated 18-month OS rates were 55.2% and 50.6%, respectively [118].

The drug has been FDA-approved first for adult patients with von Hippel–Lindau (VHL) disease, then for adult patients with advanced RCC (following a PD-1/PD-L1 inhibitor and a VEGF-TKI therapies). This is the main approval for the post-ICI/TKI setting in advanced disease.

Belzutifan is generally well tolerated; however, anemia represents a predictable on-target toxicity, likely resulting from HIF-2α inhibition of erythropoietin production. In the phase III LITESPARK-005 trial, anemia occurred in 88.3% of patients, while hypoxia occurred in approximately 15% of patients in the belzutifan arm (69% of whom requiring supplemental oxygen), including grade 3 hypoxia in about 10%, necessitating regular monitoring of hemoglobin levels and oxygen saturation during treatment [118].

### 4.2. Targeting Other Metabolic Pathways

Besides belzutifan, strategies targeting the other metabolic pathways have been studied or are in clinical trials. Table 1 summarizes the current status of these studies, some of which are promising preclinical studies or are clinical trials in other cancers but showing promise. These can be considered in the ccRCC setting. Some have not shown promising clinical results but may worth considering using different trial designs.

**Table 1:** Summary of potential therapeutics against the dysregulated metabolic pathways.

Class	Target	Example Agents	ccRCC Clinical Status	Limitations
**Glutamine** **metabolism**	Glutaminase	Telaglenastat/CB-839	(1) Phase 1b trial (NCT02071862) showed activity with everolimus/cabozantinib in ccRCC [119], but (2) Phase 2/3 CANTATA trial did not improve PFS in combination therapy [120].	(1) A small, non-randomized trial; no validated biomarker selection. (2) Adding telaglenastat to cabozantinib did not improve outcome in an unselected population; no monotherapy arm.
**Lactate** **import/export**	MCT1	AZD3965	First-in-human trial in advanced solid tumors/lymphoma, not in ccRCC; acceptable safety, target engagement, and evidence of lactate transport inhibition [121].	A safety study, not an efficacy study; AZD3965 is a MCT1 inhibitor, but ccRCC expresses high MCT4.
	MCT1	BAY-8002	Preclinical study; inhibits MCT1-mediated lactate transport, suppresses tumor cell proliferation, and shows antitumor activity in MCT1-dependent models. Not in ccRCC [122].	A preclinical study; BAY-8002 is a MCT1 inhibitor, but ccRCC expresses high MCT4.
	MCT4	MCT4 inhibitor syrosingopine	Preclinical study; causes lactate accumulation in ccRCC cells and reduced viability [123].	Preclinical study. Strong dependence on combination therapy with metformin and phenformin.
**Lactate** **production**	LDHA	LDHA inhibition (represented by experimental inhibitors such as FX11 and GNE-140)	Preclinical study. Small-molecule inhibitors such as FX11 and GNE-140 demonstrate antitumor activity [124,125]; not in ccRCC.	Preclinical study; not ccRCC-focused; cytostatic rather than cytotoxic activity (FX11); development of resistance.
**Hypoxia** **pathway** **therapy**	HIF-2α	Belzutifan	Approved after PD-1/PD-L1 + VEGF therapy; improved PFS/ORR vs everolimus in LITESPARK-005 [117,118].	Resistance commonly develops; anemia a common on-target toxicity; can develop hypoxemia, necessitating monitoring of oxygen saturation during therapy.
**Tryptophan–kynurenine pathway**	IDO1	Epacadostat	Phase 3 KEYNOTE-679/ECHO-302 trial (NCT03260894) of pembrolizumab plus epacadostat vs. sunitinib or pazopanib in metastatic ccRCC; terminated early [126].	Did not reach originally planned statistical maturity; no pembrolizumab-alone control arm; no marker-selected for IDO1 expression, serum kynurenine, Kyn/Trp ratio, or AhR activation; incomplete suppression of Kyn level.
**Kynurenine–AhR axis**	AhR	IK-175	Early clinical development in solid tumors/urothelial cancer (NCT04200963); no established ccRCC role [86,127].	IK-175: Phase 1 study, no ccRCC cohort; limited improvement over nivolumab monotherapy.
**Arginine immune checkpoint**	ARG1/ARG2	INCB001158	Phase 1 solid-tumor trial (NCT02903914); no ccRCC-specific use [128].	No ccRCC cohort; evidence of arginase inhibition but antitumor activity limited; no biomarker selection such as ARG1 expression.
	Circulating Arg	ADI-PEG20 (Pegylated arginine deiminase)	Various clinical trials in different cancers but not in ccRCC [129,130].	Improved survival in some cancers [129,130]; no trials in ccRCC; may exacerbate systemic Arg deficiency; neutralizing anti-ADI antibodies can develop.
**Adenosine** **generation**	CD73/CD39	Oleclumab (anti-CD73), anti-CD39 antibodies	Early solid-tumor trials NCT02503774; not in ccRCC [131].	No ccRCC; modest monotherapy activity.
**Adenosine** **pathway**	A2AR	Ciforadenant/CPI-444	Phase 1 RCC trial (NCT02655822) showed safety and modest activity alone or with atezolizumab [132].	Small patient size; patients had advanced, heavily pretreated RCC; modest efficacy likely due to receptor redundancy (A2BR).

### 4.3. Combination Therapy

In summary, the modest clinical success in ccRCC so far reflects the challenge of targeting downstream metabolic stress separately without addressing the upstream *VHL* deficiency/HIF driver. Standard care therefore remains ICI+TKI combinations, with belzutifan serving as the key metabolic-add-on option.

On the other hand, single-agent belzutifan works well against sporadic ccRCC but is not superior over existing therapies in OS. Belzutifan therapy may leave residual Kyn/Arg stress or adenosine toxicity in many patients, and the on-target bystander effects are still of concern. In such cases, adding other agents targeting the downstream metabolic pathways may enhance response durability with reduced side effects. For example, adenosine pathway inhibition benefits from targeting a highly immunosuppressive metabolite (adenosine) produced via CD39/CD73 in the TME, not inside the cells, with low toxicity.

Table 2 summarizes potential combination therapies based on the studies of metabolic pathways described above.

### 4.4. Metabolism-Targeted Therapies—Current Status

The modest clinical efficacy observed in many metabolism-targeted therapies likely results from multiple biological and therapeutic challenges. Tumor metabolic pathways are highly interconnected, functionally redundant, and capable of rapid adaptation. Consequently, inhibition of a single metabolic pathway often triggers compensatory activation of alternative nutrient utilization or immunosuppressive metabolic programs. Emerging evidence further indicates that ccRCC exhibits remarkable metabolic plasticity, enabling dynamic metabolic reprogramming in response to therapeutic stress [13]. In addition, most clinical trials have enrolled heavily pretreated patients with advanced disease, in whom T cells may have already progressed to a terminally exhausted state that is difficult to reverse. Under these conditions, prolonged immunometabolic stress may not merely suppress transient T-cell activation but instead reinforce stable dysfunctional states through activation of proteotoxic and integrated stress-response pathways [133].

Most clinical trials have not incorporated biomarker-guided patient stratification despite the marked metabolic heterogeneity of ccRCC. Table 3 summarizes potential biomarkers for patient selection and treatment monitoring. Recent multiomic analyses have identified metabolically adaptive ccRCC subpopulations with enhanced nutrient acquisition and aggressive behavior, suggesting that in-treatment metabolic evolution contributes to therapeutic resistance and tumor recurrence [134]. Furthermore, limited intratumoral drug penetration and steep metabolic gradients within hypoxic regions may reduce effective target inhibition despite adequate systemic drug exposure. Collectively, these findings indicate that immune evasion in ccRCC is driven not by a single pathway but by a dynamic, interconnected immunometabolic network.

### 4.5. Unresolved Issues and Future Perspectives

One major unresolved question is whether terminally exhausted T cells in advanced tumors can be fully reactivated even after correction of metabolic dysfunction within the TME. Chronic exposure to hypoxia, nutrient deprivation, and toxic metabolic byproducts may induce broader integrated stress-response programs in tumor-infiltrating lymphocytes, thereby stabilizing terminal exhaustion states and limiting the reversibility of T-cell dysfunction. Future investigations integrating spatial transcriptomics, metabolomics, longitudinal clinical profiling, and single-cell analyses will be critical for identifying therapeutically actionable metabolic vulnerabilities and optimizing patient selection for combination-based treatment strategies.

## 5. Conclusions

The major metabolic pathways reprogrammed in ccRCC are summarized in Table 4. Although immune checkpoint inhibitors have transformed the treatment of ccRCC, durable clinical responses remain limited, highlighting persistent mechanisms of immunometabolic resistance. Collectively, current evidence identifies tumor metabolism as a central regulator of immune evasion and a promising therapeutic target. Future clinical studies should prioritize biomarker-guided evaluation of combination strategies targeting multiple immunometabolic pathways together with immune checkpoint blockade rather than single-pathway inhibition alone.

## Figures and Tables

**Table 2:** Potential combination therapeutics.

Strategy	Biological Rationale	Biomarker-Selected Population	Advantage	Potential Limitations
Belzutifan + AhR inhibitor	Belzutifan addresses the upstream driver, while an AhR inhibitor addresses the downstream Kyn escape pathway	Patients with: elevated serum Kyn/Trp ratio high tumor IDO1/TDO2 expression	Uses clinically advanced agents; mechanistically coherent; some biomarkers already available	No validated AhR biomarker yet
Belzutifan + MCT4 inhibitor	Belzutifan addresses the upstream driver, while a MCT4 inhibitor prevents lactate secretion and potentially causing toxicity in ccRCC cells	Patients with: high MCT4 IHC high plasma lactate CAIX expression or other HIF pathway signature	Strong preclinical ccRCC-specific evidence	No mature clinical-stage MCT4 inhibitor currently exists
Belzutifan + A2AR/CD73 blockade	Belzutifan may reduce adenosine generation, while A2AR/CD73 blockade prevents downstream signaling	Patients with: CD73-high tumors adenosine signature (AdenoSig) high tumors	Adenosine is a validated metabolic checkpoint in ccRCC; targeting a immunosuppressive metabolite in the TME, with low toxicity	Adenosine receptor redundancy

**Table 3:** Suggested biomarkers of the metabolic reprogramming pathways.

Metabolic Pathways	Potential Markers	Clinical Applications	References
**Lactate acidosis**	Tissue IHC: MCT1 and MCT4	High MCT1 or MCT4 expression independently predicted worse OS and PFS of ccRCC patients. In patients treated with VEGFR inhibitors, high MCT4 predicted shorter PFS.	[135]
	Liquid biopsy: Serum LDH (traditionally prognostic, but now tested as a surrogate for global TME acidity and tumor burden).	Baseline serum LDH was included in the original Memorial Sloan Kettering Cancer Center (MSKCC) prognostic model, where elevated levels identified poor-risk metastatic RCC. Replaced by neutrophilia and thrombocytosis in the International Metastatic RCC Database Consortium (IMDC) model, but elevated LDH remains an adverse prognostic biomarker for high tumor burden and enhanced glycolysis.	[136,137]
**Arginine depletion**	Tissue: NanoString nCounter PanCancer IO 360 Panel and multiplexed immuno-fluorescence staining for ARG1 in ccRCC tumor tissue.	Tissue ARG1 as an outcome predictor: In toripalimab combined with axitinib treatment in ccRCC patients (NCT04118855). Responders exhibited lower pretreatment expression of ARG1.	[138]
	Liquid biopsy: Mass spec-based determination of a low plasma L-Arg to L-Orn ratio or elevated circulating/exosomal ARG1 protein concentrations.	In studies involving dual arginase inhibitors combined with ICIs for metastatic solid tumors, tracking the correction of the plasma L-Arg deficit to confirm “on-target” metabolic reversal in vivo. Not ccRCC-specific.	[128]
**Tryptophan depletion & Kyn-mediated T-cell exhaustion**	Tissue: IHC or transcriptomic evaluation of IDO1 and TDO2 expression (spatial presence in tumor endothelial cells or immune infiltrates).	Endothelial IDO1 for Nivolumab stratification: In metastatic RCC patients undergoing second-line ICI therapy with Nivolumab, IDO1 expression is a superior predictive marker over traditional PD-L1 scoring. High IDO1 expression localized in tumor endothelial cells (>10% by IHC) correlates with a 100% ORR and a superior PFS compared.	[139]
	Liquid biopsy: Longitudinal serum samples from three independent clinical trials of nivolumab: two Phase 1 trials, and a Phase 3 CheckMate 025 trial for metastatic RCC, comparing nivolumab to everolimus.	Serum Kyn levels and Kyn/Trp ratio as outcome predictors: Increased Kyn/Trp ratio indicates an adaptive resistance mechanism associated with worse OS.	[140]
	Liquid biopsy: Quantification of the plasma Kyn/Trp ratio using liquid chromatography-mass spectrometry.	Kyn/Trp ratio and ARG1 expression as sunitinib outcome predictors: In retrospective, multicenter trials evaluating first-line sunitinib efficacy in advanced ccRCC, elevated Kyn/Trp ratio, along with higher serum ARG1 concentrations, serve as significant independent predictors of shorter PFS and OS.	[141]
**Adenosine-driven** **immune suppression**	Tissue: RNA from RCC tumor tissues using NanoSptring PanCancer Immune Profiling Panel.	AdenoSig as an outcome predicter: In phase 1 study of the first-in-class A2A receptor antagonist ciforadenant for RCC patients, clinical benefit was associated with a pretreatment adenosine-regulated gene expression signature (AdenoSig: IL1β, PTGS2, and CXCL1, 2, 3, 5, 6, 8).	[132]

**Table 4:** Summary of dysregulated metabolic pathways that promote immune suppression.

Pathway	Cause	Result	Pathological Effect	Therapeutic Targets/ccRCC Clinical Status (y/n)	Outcomes and Limitations
**Acidosis** [36,37]	Increased glycolysis	Lactic acid accumulation in the TME	Acidification resulting in loss of Teffs activity and activation of Tregs	1. MCT1, 4/n 2. LDHA/n 3. HIF-2α/y	1. Preclinical studies; not ccRCC-focused; MCT1 and MCT4 having redundant functions [121,122]; dependent on combination with metformin and phenformin [123]. 2. Preclinical study; showing cytostatic rather than cytotoxic activity; development of resistance [124,125]. 3. FDA-approved; on-target side effect [117,118].
**Reductive carboxylation** [32,34,119]	Reduced TCA cycle and increased metabolism of glutamine	Increased AcCoA and lipid synthesis	Lipid accumulation	Glutaminase/y (ccRCC clinical trials)	Failure to improve PFS in phase 3 randomized ccRCC trial [120].
**Arginine depletion** [57,60]	Downregulation of urea cycle enzymes ASS1 and ASL	Arg auxotrophy in cancer cells; Arg deficiency in the TME	Increased exhaustion of effector cells	Depleting circulating Arg by ADI-PEG20/n (Various clinical trials; not in ccRCC)	Demonstrated improved survival in some cancers [129,130]; no ccRCC; may exacerbate systemic Arg deficiency; ADI is a bacterial enzyme, neutralizing antibodies can develop.
	Increased expression of ARG1 in myeloid cells	Arg deficiency in the TME	Increased exhaustion of effector cells and activation of TAM-M2 and MDSCs	ARG1 inhibitor/n (phase 1 solid-tumor trial; no ccRCC)	Evidence of arginase inhibition but antitumor activity limited; no published ccRCC-specific clinical benefit [128].
**Tryptophan depletion** [66,68,71]	Upregulation of IDO1 and TDO	Increased Trp uptake from TME; increased production of Kyn	Depletion of Trp in the TME; inactivation of effector cells	IDO1 inhibitor/y (Clinical trial in ccRCC)	Did not reach originally planned statistical maturity; incomplete suppression of Kyn accumulation [126].
**Kyn-mediated T cell exhaustion** [72,81,82,83]	Upregulation of Kyn-AhR signaling	Increased Kyn production and levels of Kyn in TME	Increased exhaustion of Teffs; activation of Tregs; increased activation of tolerogenic DC and TAM-M2	AhR inhibitor/n (Clinical trial on urothelial carcinoma)	Small phase 1 trial; limited improvement over nivolumab monotherapy; no published ccRCC-specific clinical benefit [86,127].
**Adenosine-driven immune suppression** [89,90,91]	Increased ATP release and conversion to adenosine by CD73 and CD39	Induce A2AR and A2BR receptor signaling in various immune cells	Induce TAM-M2 and MDSC; reduce antigen presentation in macrophages and DCs; induce Treg	A2AR inhibitor/y (phase 1 clinical trial in RCC)	Small patient size; modest efficacy likely due to adenosine receptor redundancy [132].
				CD39 antibody/n (Early solid-tumor trials; not in ccRCC)	Modest monotherapy activity; no established efficacy in ccRCC [131].

## Data Availability

No new data were created or analyzed in this study. Data sharing is not applicable to this article.

## References

[1] Hsieh J.J., Purdue M.P., Signoretti S., Swanton C., Albiges L., Schmidinger M., Heng D.Y., Larkin J., Ficarra V. (2017). Renal cell carcinoma. Nat. Rev. Dis. Primers.

[2] Padala S.A., Barsouk A., Thandra K.C., Saginala K., Mohammed A., Vakiti A., Rawla P., Barsouk A. (2020). Epidemiology of Renal Cell Carcinoma. World J. Oncol..

[3] Bergers G., Hanahan D. (2008). Modes of resistance to anti-angiogenic therapy. Nat. Rev. Cancer.

[4] Liu Z.L., Chen H.H., Zheng L.L., Sun L.P., Shi L. (2023). Angiogenic signaling pathways and anti-angiogenic therapy for cancer. Signal Transduct. Target Ther..

[5] Motzer R.J., Escudier B., McDermott D.F., George S., Hammers H.J., Srinivas S., Tykodi S.S., Sosman J.A., Procopio G., Plimack E.R. (2015). Nivolumab versus Everolimus in Advanced Renal-Cell Carcinoma. N. Engl. J. Med..

[6] Topalian S.L., Hodi F.S., Brahmer J.R., Gettinger S.N., Smith D.C., McDermott D.F., Powderly J.D., Carvajal R.D., Sosman J.A., Atkins M.B. (2012). Safety, activity, and immune correlates of anti-PD-1 antibody in cancer. N. Engl. J. Med..

[7] Motzer R.J., Tannir N.M., McDermott D.F., Aren Frontera O., Melichar B., Choueiri T.K., Plimack E.R., Barthelemy P., Porta C., George S. (2018). Nivolumab plus Ipilimumab versus Sunitinib in Advanced Renal-Cell Carcinoma. N. Engl. J. Med..

[8] Rini B.I., Plimack E.R., Stus V., Gafanov R., Hawkins R., Nosov D., Pouliot F., Alekseev B., Soulieres D., Melichar B. (2019). Pembrolizumab plus Axitinib versus Sunitinib for Advanced Renal-Cell Carcinoma. N. Engl. J. Med..

[9] The Cancer Genome Atlas Research Network (2013). Comprehensive molecular characterization of clear cell renal cell carcinoma. Nature.

[10] Sato Y., Yoshizato T., Shiraishi Y., Maekawa S., Okuno Y., Kamura T., Shimamura T., Sato-Otsubo A., Nagae G., Suzuki H. (2013). Integrated molecular analysis of clear-cell renal cell carcinoma. Nat. Genet..

[11] Shen C., Kaelin W.G. (2013). The VHL/HIF axis in clear cell renal carcinoma. Semin. Cancer Biol..

[12] Semenza G.L. (2009). Regulation of cancer cell metabolism by hypoxia-inducible factor 1. Semin. Cancer Biol..

[13] Zhang Y., Zhang S., Sun H., Xu L. (2025). The pathogenesis and therapeutic implications of metabolic reprogramming in renal cell carcinoma. Cell Death Discov..

[14] Kim M.C., Jin Z., Kolb R., Borcherding N., Chatzkel J.A., Falzarano S.M., Zhang W. (2021). Updates on Immunotherapy and Immune Landscape in Renal Clear Cell Carcinoma. Cancers.

[15] Sobottka B., Vetter V., Banaei-Esfahani A., Nowak M., Lorch A., Sirek A., Mertz K.D., Brunelli M., Berthold D., de Leval L. (2024). Immune Phenotype-Genotype Associations in Primary Clear Cell Renal Cell Carcinoma and Matched Metastatic Tissue. Mod. Pathol..

[16] Au L., Hatipoglu E., Robert de Massy M., Litchfield K., Beattie G., Rowan A., Schnidrig D., Thompson R., Byrne F., Horswell S. (2021). Determinants of anti-PD-1 response and resistance in clear cell renal cell carcinoma. Cancer Cell.

[17] Borcherding N., Vishwakarma A., Voigt A.P., Bellizzi A., Kaplan J., Nepple K., Salem A.K., Jenkins R.W., Zakharia Y., Zhang W. (2021). Mapping the immune environment in clear cell renal carcinoma by single-cell genomics. Commun. Biol..

[18] Chevrier S., Levine J.H., Zanotelli V.R.T., Silina K., Schulz D., Bacac M., Ries C.H., Ailles L., Jewett M.A.S., Moch H. (2017). An Immune Atlas of Clear Cell Renal Cell Carcinoma. Cell.

[19] Braun D.A., Street K., Burke K.P., Cookmeyer D.L., Denize T., Pedersen C.B., Gohil S.H., Schindler N., Pomerance L., Hirsch L. (2021). Progressive immune dysfunction with advancing disease stage in renal cell carcinoma. Cancer Cell.

[20] Dieterich L.C., Tacconi C., Ducoli L., Detmar M. (2022). Lymphatic vessels in cancer. Physiol. Rev..

[21] Donnan M.D., Kenig-Kozlovsky Y., Quaggin S.E. (2021). The lymphatics in kidney health and disease. Nat. Rev. Nephrol..

[22] Russell P.S., Hong J., Windsor J.A., Itkin M., Phillips A.R.J. (2019). Renal Lymphatics: Anatomy, Physiology, and Clinical Implications. Front. Physiol..

[23] Dufies M., Giuliano S., Ambrosetti D., Claren A., Ndiaye P.D., Mastri M., Moghrabi W., Cooley L.S., Ettaiche M., Chamorey E. (2017). Sunitinib Stimulates Expression of VEGFC by Tumor Cells and Promotes Lymphangiogenesis in Clear Cell Renal Cell Carcinomas. Cancer Res..

[24] Dieterich L.C., Ikenberg K., Cetintas T., Kapaklikaya K., Hutmacher C., Detmar M. (2017). Tumor-Associated Lymphatic Vessels Upregulate PDL1 to Inhibit T-Cell Activation. Front. Immunol..

[25] Li X., Liu P., Li M., Zhou Y., Li T., Yang T., Dong J., Fu C., Zhu Y., Chen L. (2026). Combining spatial and single-cell transcriptome data to analyze tertiary lymphoid structures in clear cell renal cell carcinoma reveals prognostic biomarkers. J. Transl. Med..

[26] Xu W., Lu J., Liu W.R., Anwaier A., Wu Y., Tian X., Su J.Q., Qu Y.Y., Yang J., Zhang H. (2023). Heterogeneity in tertiary lymphoid structures predicts distinct prognosis and immune microenvironment characterizations of clear cell renal cell carcinoma. J. Immunother. Cancer.

[27] Linehan W.M., Schmidt L.S., Crooks D.R., Wei D., Srinivasan R., Lang M., Ricketts C.J. (2019). The Metabolic Basis of Kidney Cancer. Cancer Discov..

[28] Hakimi A.A., Reznik E., Lee C.H., Creighton C.J., Brannon A.R., Luna A., Aksoy B.A., Liu E.M., Shen R., Lee W. (2016). An Integrated Metabolic Atlas of Clear Cell Renal Cell Carcinoma. Cancer Cell.

[29] Chen C., Pore N., Behrooz A., Ismail-Beigi F., Maity A. (2001). Regulation of glut1 mRNA by hypoxia-inducible factor-1. Interaction between H-ras and hypoxia. J. Biol. Chem..

[30] Hayashi M., Sakata M., Takeda T., Yamamoto T., Okamoto Y., Sawada K., Kimura A., Minekawa R., Tahara M., Tasaka K. (2004). Induction of glucose transporter 1 expression through hypoxia-inducible factor 1alpha under hypoxic conditions in trophoblast-derived cells. J. Endocrinol..

[31] Guo D., Meng Y., Jiang X., Lu Z. (2023). Hexokinases in cancer and other pathologies. Cell Insight.

[32] Metallo C.M., Gameiro P.A., Bell E.L., Mattaini K.R., Yang J., Hiller K., Jewell C.M., Johnson Z.R., Irvine D.J., Guarente L. (2012). Reductive glutamine metabolism by IDH1 mediates lipogenesis under hypoxia. Nature.

[33] Kim J.W., Gao P., Liu Y.C., Semenza G.L., Dang C.V. (2007). Hypoxia-inducible factor 1 and dysregulated c-Myc cooperatively induce vascular endothelial growth factor and metabolic switches hexokinase 2 and pyruvate dehydrogenase kinase 1. Mol. Cell Biol..

[34] Gameiro P.A., Yang J., Metelo A.M., Perez-Carro R., Baker R., Wang Z., Arreola A., Rathmell W.K., Olumi A., Lopez-Larrubia P. (2013). In vivo HIF-mediated reductive carboxylation is regulated by citrate levels and sensitizes VHL-deficient cells to glutamine deprivation. Cell Metab..

[35] Kaelin W.G. (2008). The von Hippel-Lindau tumour suppressor protein: O2 sensing and cancer. Nat. Rev. Cancer.

[36] Siska P.J., Beckermann K.E., Mason F.M., Andrejeva G., Greenplate A.R., Sendor A.B., Chiang Y.J., Corona A.L., Gemta L.F., Vincent B.G. (2017). Mitochondrial dysregulation and glycolytic insufficiency functionally impair CD8 T cells infiltrating human renal cell carcinoma. JCI Insight.

[37] Reinfeld B.I., Rathmell W.K., Kim T.K., Rathmell J.C. (2022). The therapeutic implications of immunosuppressive tumor aerobic glycolysis. Cell Mol. Immunol..

[38] Chang C.H., Curtis J.D., Maggi L.B., Faubert B., Villarino A.V., O’Sullivan D., Huang S.C., van der Windt G.J., Blagih J., Qiu J. (2013). Posttranscriptional control of T cell effector function by aerobic glycolysis. Cell.

[39] Dimeloe S., Burgener A.V., Grahlert J., Hess C. (2017). T-cell metabolism governing activation, proliferation and differentiation; a modular view. Immunology.

[40] Kumagai S., Koyama S., Itahashi K., Tanegashima T., Lin Y.T., Togashi Y., Kamada T., Irie T., Okumura G., Kono H. (2022). Lactic acid promotes PD-1 expression in regulatory T cells in highly glycolytic tumor microenvironments. Cancer Cell.

[41] Watson M.J., Vignali P.D.A., Mullett S.J., Overacre-Delgoffe A.E., Peralta R.M., Grebinoski S., Menk A.V., Rittenhouse N.L., DePeaux K., Whetstone R.D. (2021). Metabolic support of tumour-infiltrating regulatory T cells by lactic acid. Nature.

[42] Angelin A., Gil-de-Gomez L., Dahiya S., Jiao J., Guo L., Levine M.H., Wang Z., Quinn W.J., Kopinski P.K., Wang L. (2017). Foxp3 Reprograms T Cell Metabolism to Function in Low-Glucose, High-Lactate Environments. Cell Metab..

[43] Howie D., Cobbold S.P., Adams E., Ten Bokum A., Necula A.S., Zhang W., Huang H., Roberts D.J., Thomas B., Hester S.S. (2017). Foxp3 drives oxidative phosphorylation and protection from lipotoxicity. JCI Insight.

[44] Chandel N.S. (2021). Amino Acid Metabolism. Cold Spring Harb. Perspect. Biol..

[45] Brosnan M.E., Brosnan J.T. (2004). Renal arginine metabolism. J. Nutr..

[46] Chen G.F., Baylis C. (2010). In vivo renal arginine release is impaired throughout development of chronic kidney disease. Am. J. Physiol. Ren. Physiol..

[47] Deng H., Bloomfield V.A., Benevides J.M., Thomas G.J. (2000). Structural basis of polyamine-DNA recognition: Spermidine and spermine interactions with genomic B-DNAs of different GC content probed by Raman spectroscopy. Nucleic Acids Res..

[48] Miska J., Rashidi A., Lee-Chang C., Gao P., Lopez-Rosas A., Zhang P., Burga R., Castro B., Xiao T., Han Y. (2021). Polyamines drive myeloid cell survival by buffering intracellular pH to promote immunosuppression in glioblastoma. Sci. Adv..

[49] Dantzler W.H., Layton A.T., Layton H.E., Pannabecker T.L. (2014). Urine-concentrating mechanism in the inner medulla: Function of the thin limbs of the loops of Henle. Clin. J. Am. Soc. Nephrol..

[50] Ochocki J.D., Khare S., Hess M., Ackerman D., Qiu B., Daisak J.I., Worth A.J., Lin N., Lee P., Xie H. (2018). Arginase 2 Suppresses Renal Carcinoma Progression via Biosynthetic Cofactor Pyridoxal Phosphate Depletion and Increased Polyamine Toxicity. Cell Metab..

[51] Khare S., Kim L.C., Lobel G., Doulias P.T., Ischiropoulos H., Nissim I., Keith B., Simon M.C. (2021). ASS1 and ASL suppress growth in clear cell renal cell carcinoma via altered nitrogen metabolism. Cancer Metab..

[52] Chantranupong L., Scaria S.M., Saxton R.A., Gygi M.P., Shen K., Wyant G.A., Wang T., Harper J.W., Gygi S.P., Sabatini D.M. (2016). The CASTOR Proteins Are Arginine Sensors for the mTORC1 Pathway. Cell.

[53] Saxton R.A., Chantranupong L., Knockenhauer K.E., Schwartz T.U., Sabatini D.M. (2016). Mechanism of arginine sensing by CASTOR1 upstream of mTORC1. Nature.

[54] Wang S., Tsun Z.Y., Wolfson R.L., Shen K., Wyant G.A., Plovanich M.E., Yuan E.D., Jones T.D., Chantranupong L., Comb W. (2015). Lysosomal amino acid transporter SLC38A9 signals arginine sufficiency to mTORC1. Science.

[55] Groth C., Hu X., Weber R., Fleming V., Altevogt P., Utikal J., Umansky V. (2019). Immunosuppression mediated by myeloid-derived suppressor cells (MDSCs) during tumour progression. Br. J. Cancer.

[56] Li K., Shi H., Zhang B., Ou X., Ma Q., Chen Y., Shu P., Li D., Wang Y. (2021). Myeloid-derived suppressor cells as immunosuppressive regulators and therapeutic targets in cancer. Signal Transduct. Target Ther..

[57] Fletcher M., Ramirez M.E., Sierra R.A., Raber P., Thevenot P., Al-Khami A.A., Sanchez-Pino D., Hernandez C., Wyczechowska D.D., Ochoa A.C. (2015). l-Arginine depletion blunts antitumor T-cell responses by inducing myeloid-derived suppressor cells. Cancer Res..

[58] Rodriguez P.C., Zea A.H., Culotta K.S., Zabaleta J., Ochoa J.B., Ochoa A.C. (2002). Regulation of T cell receptor CD3zeta chain expression by L-arginine. J. Biol. Chem..

[59] Zea A.H., Rodriguez P.C., Culotta K.S., Hernandez C.P., DeSalvo J., Ochoa J.B., Park H.J., Zabaleta J., Ochoa A.C. (2004). L-Arginine modulates CD3zeta expression and T cell function in activated human T lymphocytes. Cell Immunol..

[60] Geiger R., Rieckmann J.C., Wolf T., Basso C., Feng Y., Fuhrer T., Kogadeeva M., Picotti P., Meissner F., Mann M. (2016). L-Arginine Modulates T Cell Metabolism and Enhances Survival and Anti-tumor Activity. Cell.

[61] Munn D.H., Sharma M.D., Baban B., Harding H.P., Zhang Y., Ron D., Mellor A.L. (2005). GCN2 kinase in T cells mediates proliferative arrest and anergy induction in response to indoleamine 2,3-dioxygenase. Immunity.

[62] Chen M., Nie Z., Huang D., Gao Y., Cao H., Zheng L., Zhang S. (2022). Development of a polyamine gene expression score for predicting prognosis and treatment response in clear cell renal cell carcinoma. Front. Immunol..

[63] di Meo N.A., Lasorsa F., Rutigliano M., Loizzo D., Ferro M., Stella A., Bizzoca C., Vincenti L., Pandolfo S.D., Autorino R. (2022). Renal Cell Carcinoma as a Metabolic Disease: An Update on Main Pathways, Potential Biomarkers, and Therapeutic Targets. Int. J. Mol. Sci..

[64] Gong Z., Lin H., Wang J., Hua J., Wei J., Chen W., Luo J., Pang J., Chen X. (2025). A robust ammonia metabolism gene signature identified by machine learning predicts prognosis and immunotherapy response in clear cell renal cell carcinoma. Front. Oncol..

[65] Zhang Y., Narayanan S.P., Mannan R., Raskind G., Wang X., Vats P., Su F., Hosseini N., Cao X., Kumar-Sinha C. (2021). Single-cell analyses of renal cell cancers reveal insights into tumor microenvironment, cell of origin, and therapy response. Proc. Natl. Acad. Sci. USA.

[66] Opitz C.A., Somarribas Patterson L.F., Mohapatra S.R., Dewi D.L., Sadik A., Platten M., Trump S. (2020). The therapeutic potential of targeting tryptophan catabolism in cancer. Br. J. Cancer.

[67] Yusri K., Jose S., Vermeulen K.S., Tan T.C.M., Sorrentino V. (2025). The role of NAD(+) metabolism and its modulation of mitochondria in aging and disease. npj Metab. Health Dis..

[68] Seo S.K., Kwon B. (2023). Immune regulation through tryptophan metabolism. Exp. Mol. Med..

[69] Fagny M., Platig J., Kuijjer M.L., Lin X., Quackenbush J. (2020). Nongenic cancer-risk SNPs affect oncogenes, tumour-suppressor genes, and immune function. Br. J. Cancer.

[70] Hennequart M., Pilotte L., Cane S., Hoffmann D., Stroobant V., Plaen E., Van den Eynde B.J. (2017). Constitutive IDO1 Expression in Human Tumors Is Driven by Cyclooxygenase-2 and Mediates Intrinsic Immune Resistance. Cancer Immunol. Res..

[71] Munn D.H., Mellor A.L. (2016). IDO in the Tumor Microenvironment: Inflammation, Counter-Regulation, and Tolerance. Trends Immunol..

[72] Joisten N., Ruas J.L., Braidy N., Guillemin G.J., Zimmer P. (2021). The kynurenine pathway in chronic diseases: A compensatory mechanism or a driving force?. Trends Mol. Med..

[73] Collins B.E., Smith B.A., Bengtson P., Paulson J.C. (2006). Ablation of CD22 in ligand-deficient mice restores B cell receptor signaling. Nat. Immunol..

[74] Fallarino F., Grohmann U., You S., McGrath B.C., Cavener D.R., Vacca C., Orabona C., Bianchi R., Belladonna M.L., Volpi C. (2006). The combined effects of tryptophan starvation and tryptophan catabolites down-regulate T cell receptor zeta-chain and induce a regulatory phenotype in naive T cells. J. Immunol..

[75] de Candia P., Procaccini C., Russo C., Lepore M.T., Matarese G. (2022). Regulatory T cells as metabolic sensors. Immunity.

[76] Halaby M.J., Hezaveh K., Lamorte S., Ciudad M.T., Kloetgen A., MacLeod B.L., Guo M., Chakravarthy A., Medina T.D.S., Ugel S. (2019). GCN2 drives macrophage and MDSC function and immunosuppression in the tumor microenvironment. Sci. Immunol..

[77] Pichler R., Siska P.J., Tymoszuk P., Martowicz A., Untergasser G., Mayr R., Weber F., Seeber A., Kocher F., Barth D.A. (2023). A chemokine network of T cell exhaustion and metabolic reprogramming in renal cell carcinoma. Front. Immunol..

[78] Tang C., Xie A.X., Liu E.M., Kuo F., Kim M., DiNatale R.G., Golkaram M., Chen Y.B., Gupta S., Motzer R.J. (2023). Immunometabolic coevolution defines unique microenvironmental niches in ccRCC. Cell Metab..

[79] Liu Y., Liang X., Dong W., Fang Y., Lv J., Zhang T., Fiskesund R., Xie J., Liu J., Yin X. (2018). Tumor-Repopulating Cells Induce PD-1 Expression in CD8(+) T Cells by Transferring Kynurenine and AhR Activation. Cancer Cell.

[80] Campesato L.F., Budhu S., Tchaicha J., Weng C.H., Gigoux M., Cohen I.J., Redmond D., Mangarin L., Pourpe S., Liu C. (2020). Blockade of the AHR restricts a Treg-macrophage suppressive axis induced by L-Kynurenine. Nat. Commun..

[81] Mezrich J.D., Fechner J.H., Zhang X., Johnson B.P., Burlingham W.J., Bradfield C.A. (2010). An interaction between kynurenine and the aryl hydrocarbon receptor can generate regulatory T cells. J. Immunol..

[82] Rad Pour S., Morikawa H., Kiani N.A., Yang M., Azimi A., Shafi G., Shang M., Baumgartner R., Ketelhuth D.F.J., Kamleh M.A. (2019). Exhaustion of CD4+ T-cells mediated by the Kynurenine Pathway in Melanoma. Sci. Rep..

[83] Lucarelli G., Rutigliano M., Ferro M., Giglio A., Intini A., Triggiano F., Palazzo S., Gigante M., Castellano G., Ranieri E. (2017). Activation of the kynurenine pathway predicts poor outcome in patients with clear cell renal cell carcinoma. Urol. Oncol..

[84] Wu Z., Wang H., Zheng Z., Lin Y., Bian L., Geng H., Huang X., Zhu J., Jing H., Zhang Y. (2025). IDO1 inhibition enhances CLDN18.2-CAR-T cell therapy in gastrointestinal cancers by overcoming kynurenine-mediated metabolic suppression in the tumor microenvironment. J. Transl. Med..

[85] Yang Q., Hao J., Chi M., Wang Y., Xin B., Huang J., Lu J., Li J., Sun X., Li C. (2022). Superior antitumor immunotherapy efficacy of kynureninase modified CAR-T cells through targeting kynurenine metabolism. Oncoimmunology.

[86] McGovern K., Castro A.C., Cavanaugh J., Coma S., Walsh M., Tchaicha J., Syed S., Natarajan P., Manfredi M., Zhang X.M. (2022). Discovery and Characterization of a Novel Aryl Hydrocarbon Receptor Inhibitor, IK-175, and Its Inhibitory Activity on Tumor Immune Suppression. Mol. Cancer Ther..

[87] Martins I., Wang Y., Michaud M., Ma Y., Sukkurwala A.Q., Shen S., Kepp O., Metivier D., Galluzzi L., Perfettini J.L. (2014). Molecular mechanisms of ATP secretion during immunogenic cell death. Cell Death Differ..

[88] Aymeric L., Apetoh L., Ghiringhelli F., Tesniere A., Martins I., Kroemer G., Smyth M.J., Zitvogel L. (2010). Tumor cell death and ATP release prime dendritic cells and efficient anticancer immunity. Cancer Res..

[89] Yegutkin G.G. (2008). Nucleotide- and nucleoside-converting ectoenzymes: Important modulators of purinergic signalling cascade. Biochim. Biophys. Acta.

[90] Allard B., Longhi M.S., Robson S.C., Stagg J. (2017). The ectonucleotidases CD39 and CD73: Novel checkpoint inhibitor targets. Immunol. Rev..

[91] Synnestvedt K., Furuta G.T., Comerford K.M., Louis N., Karhausen J., Eltzschig H.K., Hansen K.R., Thompson L.F., Colgan S.P. (2002). Ecto-5′-nucleotidase (CD73) regulation by hypoxia-inducible factor-1 mediates permeability changes in intestinal epithelia. J. Clin. Investig..

[92] Tripathi A., Lin E., Xie W., Flaifel A., Steinharter J.A., Stern Gatof E.N., Bouchard G., Fleischer J.H., Martinez-Chanza N., Gray C. (2020). Prognostic significance and immune correlates of CD73 expression in renal cell carcinoma. J. Immunother. Cancer.

[93] de Lera Ruiz M., Lim Y.H., Zheng J. (2014). Adenosine A2A receptor as a drug discovery target. J. Med. Chem..

[94] Ohta A., Sitkovsky M. (2001). Role of G-protein-coupled adenosine receptors in downregulation of inflammation and protection from tissue damage. Nature.

[95] Ohta A., Gorelik E., Prasad S.J., Ronchese F., Lukashev D., Wong M.K., Huang X., Caldwell S., Liu K., Smith P. (2006). A2A adenosine receptor protects tumors from antitumor T cells. Proc. Natl. Acad. Sci. USA.

[96] Vecchio E.A., Tan C.Y., Gregory K.J., Christopoulos A., White P.J., May L.T. (2016). Ligand-Independent Adenosine A2B Receptor Constitutive Activity as a Promoter of Prostate Cancer Cell Proliferation. J. Pharmacol. Exp. Ther..

[97] Kamai T., Kijima T., Tsuzuki T., Nukui A., Abe H., Arai K., Yoshida K.I. (2021). Increased expression of adenosine 2A receptors in metastatic renal cell carcinoma is associated with poorer response to anti-vascular endothelial growth factor agents and anti-PD-1/Anti-CTLA4 antibodies and shorter survival. Cancer Immunol. Immunother..

[98] Yang L., Zhang Y., Yang L. (2024). Adenosine signaling in tumor-associated macrophages and targeting adenosine signaling for cancer therapy. Cancer Biol. Med..

[99] Cekic C., Day Y.J., Sag D., Linden J. (2014). Myeloid expression of adenosine A2A receptor suppresses T and NK cell responses in the solid tumor microenvironment. Cancer Res..

[100] Imani S., Jabbarzadeh Kaboli P., Babaeizad A., Maghsoudloo M. (2025). Neoantigen mRNA vaccines and A(2)A receptor antagonism: A strategy to enhance T cell immunity. Hum. Vaccin. Immunother..

[101] Wang L., Zhang J., Zhang W., Zheng M., Guo H., Pan X., Li W., Yang B., Ding L. (2024). The inhibitory effect of adenosine on tumor adaptive immunity and intervention strategies. Acta Pharm. Sin. B.

[102] Schmiel S.E., Yang J.A., Jenkins M.K., Mueller D.L. (2017). Cutting Edge: Adenosine A2a Receptor Signals Inhibit Germinal Center T Follicular Helper Cell Differentiation during the Primary Response to Vaccination. J. Immunol..

[103] Iannone R., Miele L., Maiolino P., Pinto A., Morello S. (2013). Blockade of A2b adenosine receptor reduces tumor growth and immune suppression mediated by myeloid-derived suppressor cells in a mouse model of melanoma. Neoplasia.

[104] Clayton A., Al-Taei S., Webber J., Mason M.D., Tabi Z. (2011). Cancer exosomes express CD39 and CD73, which suppress T cells through adenosine production. J. Immunol..

[105] Vijayan D., Young A., Teng M.W.L., Smyth M.J. (2017). Targeting immunosuppressive adenosine in cancer. Nat. Rev. Cancer.

[106] Takeda M., Akamatsu S., Kita Y., Goto T., Kobayashi T. (2023). The Roles of Extracellular Vesicles in the Progression of Renal Cell Carcinoma and Their Potential for Future Clinical Application. Nanomaterials.

[107] Rohde D., Wiesner C., Graf D., Wolff J., Fuzesi L., Jakse G. (2000). Interstitial fluid pressure is increased in renal cell carcinoma xenografts. Urol. Res..

[108] Milosevic M.F., Fyles A.W., Hill R.P. (1999). The relationship between elevated interstitial fluid pressure and blood flow in tumors: A bioengineering analysis. Int. J. Radiat. Oncol. Biol. Phys..

[109] Heldin C.H., Rubin K., Pietras K., Ostman A. (2004). High interstitial fluid pressure—An obstacle in cancer therapy. Nat. Rev. Cancer.

[110] Wu M., Frieboes H.B., McDougall S.R., Chaplain M.A., Cristini V., Lowengrub J. (2013). The effect of interstitial pressure on tumor growth: Coupling with the blood and lymphatic vascular systems. J. Theor. Biol..

[111] Nia H.T., Munn L.L., Jain R.K. (2020). Physical traits of cancer. Science.

[112] Lobel G.P., Jiang Y., Simon M.C. (2023). Tumor microenvironmental nutrients, cellular responses, and cancer. Cell Chem. Biol..

[113] Abbott K.L., Ali A., Reinfeld B.I., Deik A., Subudhi S., Landis M.D., Hongo R.A., Young K.L., Kunchok T., Nabel C.S. (2024). Metabolite profiling of human renal cell carcinoma reveals tissue-origin dominance in nutrient availability. Elife.

[114] Jain R.K. (2005). Normalization of tumor vasculature: An emerging concept in antiangiogenic therapy. Science.

[115] Huang Y., Yuan J., Righi E., Kamoun W.S., Ancukiewicz M., Nezivar J., Santosuosso M., Martin J.D., Martin M.R., Vianello F. (2012). Vascular normalizing doses of antiangiogenic treatment reprogram the immunosuppressive tumor microenvironment and enhance immunotherapy. Proc. Natl. Acad. Sci. USA.

[116] Magnussen A.L., Mills I.G. (2021). Vascular normalisation as the stepping stone into tumour microenvironment transformation. Br. J. Cancer.

[117] Beckermann K. (2026). From prevention to progression: Can belzutifan-based strategies deliver across the RCC disease continuum?. Oncologist.

[118] Choueiri T.K., Powles T., Peltola K., de Velasco G., Burotto M., Suarez C., Ghatalia P., Iacovelli R., Lam E.T., Verzoni E. (2024). Belzutifan versus Everolimus for Advanced Renal-Cell Carcinoma. N. Engl. J. Med..

[119] Meric-Bernstam F., Tannir N.M., Iliopoulos O., Lee R.J., Telli M.L., Fan A.C., DeMichele A., Haas N.B., Patel M.R., Harding J.J. (2022). Telaglenastat Plus Cabozantinib or Everolimus for Advanced or Metastatic Renal Cell Carcinoma: An Open-Label Phase I Trial. Clin. Cancer Res..

[120] Tannir N.M., Agarwal N., Porta C., Lawrence N.J., Motzer R., McGregor B., Lee R.J., Jain R.K., Davis N., Appleman L.J. (2022). Efficacy and Safety of Telaglenastat Plus Cabozantinib vs Placebo Plus Cabozantinib in Patients With Advanced Renal Cell Carcinoma: The CANTATA Randomized Clinical Trial. JAMA Oncol..

[121] Halford S., Veal G.J., Wedge S.R., Payne G.S., Bacon C.M., Sloan P., Dragoni I., Heinzmann K., Potter S., Salisbury B.M. (2023). A Phase I Dose-escalation Study of AZD3965, an Oral Monocarboxylate Transporter 1 Inhibitor, in Patients with Advanced Cancer. Clin. Cancer Res..

[122] Quanz M., Bender E., Kopitz C., Grunewald S., Schlicker A., Schwede W., Eheim A., Toschi L., Neuhaus R., Richter C. (2018). Preclinical Efficacy of the Novel Monocarboxylate Transporter 1 Inhibitor BAY-8002 and Associated Markers of Resistance. Mol. Cancer Ther..

[123] Menig-Benzig L.S., Stuhler V., Mazzola P., Heinrich H., Hofmann U., Widmann N., Bohnert R., Neef S.K., Sauter-Meyerhoff C., Schmees C. (2025). Drug targeting of the monocarboxylate transporter MCT4 is a novel treatment strategy for metastatic ccRCC. Pharmacol. Res..

[124] Boudreau A., Purkey H.E., Hitz A., Robarge K., Peterson D., Labadie S., Kwong M., Hong R., Gao M., Del Nagro C. (2016). Metabolic plasticity underpins innate and acquired resistance to LDHA inhibition. Nat. Chem. Biol..

[125] Le A., Cooper C.R., Gouw A.M., Dinavahi R., Maitra A., Deck L.M., Royer R.E., Vander Jagt D.L., Semenza G.L., Dang C.V. (2010). Inhibition of lactate dehydrogenase A induces oxidative stress and inhibits tumor progression. Proc. Natl. Acad. Sci. USA.

[126] Lara P.N., Villanueva L., Ibanez C., Erman M., Lee J.L., Heinrich D., Lipatov O.N., Gedye C., Gokmen E., Acevedo A. (2024). A randomized, open-label, phase 3 trial of pembrolizumab plus epacadostat versus sunitinib or pazopanib as first-line treatment for metastatic renal cell carcinoma (KEYNOTE-679/ECHO-302). BMC Cancer.

[127] Aggen D.H., McKean M., Hoffman-Censits J.H., Lakhani N.J., Alhalabi O., Guancial E.A., Bashir B., Bowman I.A., Tan A., Lingaraj T. (2026). IK-175, an Oral Aryl Hydrocarbon Receptor Inhibitor, Alone and with Nivolumab in Patients with Advanced Solid Tumors and Urothelial Carcinoma. Clin. Cancer Res..

[128] Naing A., Papadopoulos K.P., Pishvaian M.J., Rahma O., Hanna G.J., Garralda E., Saavedra O., Gogov S., Kallender H., Cheng L. (2024). First-in-human phase 1 study of the arginase inhibitor INCB001158 alone or combined with pembrolizumab in patients with advanced or metastatic solid tumours. BMJ Oncol..

[129] Szlosarek P.W., Creelan B.C., Sarkodie T., Nolan L., Taylor P., Olevsky O., Grosso F., Cortinovis D., Chitnis M., Roy A. (2024). Pegargiminase Plus First-Line Chemotherapy in Patients With Nonepithelioid Pleural Mesothelioma: The ATOMIC-Meso Randomized Clinical Trial. JAMA Oncol..

[130] Chu Y.D., Lai M.W., Yeh C.T. (2023). Unlocking the Potential of Arginine Deprivation Therapy: Recent Breakthroughs and Promising Future for Cancer Treatment. Int. J. Mol. Sci..

[131] Bendell J., LoRusso P., Overman M., Noonan A.M., Kim D.W., Strickler J.H., Kim S.W., Clarke S., George T.J., Grimison P.S. (2023). First-in-human study of oleclumab, a potent, selective anti-CD73 monoclonal antibody, alone or in combination with durvalumab in patients with advanced solid tumors. Cancer Immunol. Immunother..

[132] Fong L., Hotson A., Powderly J.D., Sznol M., Heist R.S., Choueiri T.K., George S., Hughes B.G.M., Hellmann M.D., Shepard D.R. (2020). Adenosine 2A Receptor Blockade as an Immunotherapy for Treatment-Refractory Renal Cell Cancer. Cancer Discov..

[133] Wang Y., Ma A., Song N.J., Shannon A.E., Amankwah Y.S., Chen X., Wu W., Wang Z., Saadey A.A., Yousif A. (2025). Proteotoxic stress response drives T cell exhaustion and immune evasion. Nature.

[134] Hu J., Wang S.G., Hou Y., Chen Z., Liu L., Li R., Li N., Zhou L., Yang Y., Wang L. (2024). Multi-omic profiling of clear cell renal cell carcinoma identifies metabolic reprogramming associated with disease progression. Nat. Genet..

[135] Cao Y.W., Liu Y., Dong Z., Guo L., Kang E.H., Wang Y.H., Zhang W., Niu H.T. (2018). Monocarboxylate transporters MCT1 and MCT4 are independent prognostic biomarkers for the survival of patients with clear cell renal cell carcinoma and those receiving therapy targeting angiogenesis. Urol. Oncol..

[136] Motzer R.J., Mazumdar M., Bacik J., Berg W., Amsterdam A., Ferrara J. (1999). Survival and prognostic stratification of 670 patients with advanced renal cell carcinoma. J. Clin. Oncol..

[137] Heng D.Y., Xie W., Regan M.M., Warren M.A., Golshayan A.R., Sahi C., Eigl B.J., Ruether J.D., Cheng T., North S. (2009). Prognostic factors for overall survival in patients with metastatic renal cell carcinoma treated with vascular endothelial growth factor-targeted agents: Results from a large, multicenter study. J. Clin. Oncol..

[138] Huang J., Wang Y., Xu F., Wang Z., Wu G., Kong W., Cheoklong N.G., Tricard T., Wu X., Zhai W. (2024). Neoadjuvant toripalimab combined with axitinib in patients with locally advanced clear cell renal cell carcinoma: A single-arm, phase II trial. J. Immunother. Cancer.

[139] Seeber A., Klinglmair G., Fritz J., Steinkohl F., Zimmer K.C., Aigner F., Horninger W., Gastl G., Zelger B., Brunner A. (2018). High IDO-1 expression in tumor endothelial cells is associated with response to immunotherapy in metastatic renal cell carcinoma. Cancer Sci..

[140] Li H., Bullock K., Gurjao C., Braun D., Shukla S.A., Bosse D., Lalani A.A., Gopal S., Jin C., Horak C. (2019). Metabolomic adaptations and correlates of survival to immune checkpoint blockade. Nat. Commun..

[141] Quintas G., Sanmartin E., Garcia-Gimenez A., Munoz-Langa J., Collado A., Suarez C., Garcia Del Muro X., Mendez-Vidal M.J., Garcia-Sanchez J., Salvador-Coloma C. (2026). Distinct protein and metabolic profiles in patients with advanced clear-cell renal cell carcinoma treated with sunitinib: A study of the Spanish oncology genitourinary group. Front. Oncol..

